# Dataset of allele and genotype frequencies of five polymorphisms candidate genes analyzed for association with body mass index in Russian women

**DOI:** 10.1016/j.dib.2019.104962

**Published:** 2019-12-09

**Authors:** Evgeny Reshetnikov, Maria Abramova, Irina Ponomarenko, Alexey Polonikov, Irina Verzilina, Inna Sorokina, Inna Aristova, Anna Yermachenko, Volodymyr Dvornyk, Mikhail Churnosov

**Affiliations:** a Department of Medical Biological Disciplines, Belgorod State University, 308015, Belgorod, Russia; b Department of Biology, Medical Genetics and Ecology, Kursk State Medical University, 305041, Kursk, Russia; c Department of Social Epidemiology, Pierre Louis Institute of Epidemiology and Public Health, 75571, Paris, France; d Sorbonne Universités, 75320, Paris, France; e Department of Life Sciences, College of Science and General Studies, Alfaisal University, 11533, Riyadh, Saudi Arabia

**Keywords:** Single nucleotide polymorphism, Body mass index, Women, *TNNI3K*, *RBJ*, *FANCL*, *MAP2K5*, *GPRC5B*

## Abstract

Data on the allele and genotype frequencies of the five single nucleotide polymorphisms (SNPs) 5 genes - rs1514175 *TNNI3K*, rs713586 RBJ, rs887912 *FANCL*, rs2241423 *MAP2K5*, rs12444979 *GPRC5B* in Russian women are presented. Several genome-wide association studies identified these SNPs could be significant genetic markers associated with body mass index (BMI). Standard methods were used for collecting of the anthropometric characteristics (height and weight). We calculated the frequencies of alleles and genotypes of five SNPs in 5 groups: all samples, underweight (BMI<18.50), normal weight (18.50–24.99), overweight (25.00–29.99), obese (>30.00).

**Table:** Specifications Table

Subject area	*Biology*
More specific subject area	*Genetics*
Type of data	*Table*
How data was acquired	*MALDI/TOF mass spectrometry using Sequenom MassARRAY 4.0 platform (Agena Bioscience™)*
Data format	*Raw and analyzed data*
Experimental factors	*Total genomic DNA was isolated from buffy coat using the standard phenol-chloroform method.*
Experimental features	*DNA samples were genotyped using the Sequenom MassARRAY® iPLEX platform, which is based on MALDI-TOF (matrix-assisted laser desorption/ionization time-of-flight) mass spectrometry*
Data source location	*Belgorod, Russia*
Data accessibility	*The data is available with this article*

**Table:** 

**Value of the Data** • The frequencies of alleles and genotypes of rs1514175 *TNNI3K*, rs713586 RBJ, rs887912 *FANCL*, rs2241423 *MAP2K5*, and rs12444979 *GPRC5B* among Russian women with different body mass index (underweight, normal weight, overweight, obesity) vary, but do not differ significantly. • The genetic polymorphisms in *TNNI3K, RBJ, FANCL, MAP2K5, GPRC5B* genes may play a role in body mass index. • The data on the allele and genotype frequencies are an important resource for understanding genetic structure of different populations. • The data can be used to study a genetic basis of body mass index and BMI-associated multifactorial diseases (obesity, arterial hypertension, metabolic syndrome, stroke, coronary artery disease, uterine leiomyoma, and the others) in various populations.

## 1 Data

The dataset represents the raw data (supplementary Table), frequencies of alleles and genotypes for five single nucleotide polymorphisms (SNPs) of 5 genes, rs1514175 *TNNI3K*, rs713586 RBJ, rs887912 *FANCL*, rs2241423 *MAP2K5*, and rs12444979 *GPRC5B* in Russian women (Table 1). These SNPs were associated with body mass index (BMI) in previously published genome-wide and candidate gene association studies [[1], [2], [3], [4], [5], [6], [7], [8], [9], [10]]. The dataset frequencies of the SNP alleles and genotypes were divided into five groups according to the BMI of the participants: all samples, underweight (BMI<18.50), normal weight (18.50–24.99), overweight (25.00–29.99), and obese (>30.00). No significant differences in the allele and genotype frequencies between the groups with underweight (BMI<18.50), normal weight (18.50–24.99), overweight (25.00–29.99) and obese (>30.00) (p > 0.05) were determined.

**Table 1:** The frequencies of alleles and genotypes for single nucleotide polymorphisms (SNPs) rs1514175 *TNNI3K*, rs713586 RBJ, rs887912 *FANCL*, rs2241423 *MAP2K5*, rs12444979 *GPRC5B* in the sample of Russian women.

SNP genotype or allele		All (n = 716)		Body mass index								
		n	frequency	Mean, kg/m^2^	underweight (BMI<18.50) (n = 40)		normal weight (18.50–24.99) (n = 445)		overweight (25.00–29.99) (n = 159)		obese (>30.00) (n = 72)	
					n	frequency	n	frequency	n	frequency	n	frequency
rs1514175 *TNNI3K*												
CC		275	0.3841	23.84 ± 4.46	21	0.5250	173	0.3888	59	0.3710	22	0.3056
CT		331	0.4623	24.16 ± 4.37	11	0.2750	206	0.4629	70	0.4403	44	0.6111
TT		110	0.1536	23.13 ± 3.70	8	0.2000	66	0.1483	30	0.1887	6	0.0833
C		881	0.6152	–	53	0.6625	552	0.6202	188	0.5912	88	0.6111
T		551	0.3848	–	27	0.3375	338	0.3798	130	0.4088	56	0.3889
rs713586 RBJ												
TT		238	0.3324	23.61 ± 4.13	18	0.4500	146	0.3281	49	0.3082	25	0.3472
TC		340	0.4749	24.08 ± 4.65	18	0.4500	212	0.4764	73	0.4591	37	0.5139
CC		138	0.1927	23.92 ± 3.74	4	0.1000	87	0.1955	37	0.2327	10	0.1389
T		816	0.5698	–	54	0.6750	504	0.5663	171	0.5377	87	0.6042
C		616	0.4302	–	26	0.3250	386	0.4337	147	0.4623	57	0.3958
rs887912 *FANCL*												
GG		437	0.6103	23.96 ± 4.47	27	0.6750	270	0.6067	95	0.5975	45	0.6250
GA		238	0.3324	23.68 ± 4.19	12	0.3000	152	0.3416	50	0.3145	24	0.3333
AA		41	0.0573	24.11 ± 3.30	1	0.0250	23	0.0517	14	0.0880	3	0.0417
G		1112	0.7765	–	66	0.8250	692	0.7775	240	0.7547	114	0.7917
A		320	0.2235	–	14	0.1750	198	0.2225	78	0.2453	30	0.2083
rs2241423 *MAP2K5*												
GG	474		0.6620	23.81 ± 4.11	27	0.6750	288	0.6471	115	0.7233	44	0.6111
GA	213		0.2975	23.93 ± 4.65	11	0.2750	141	0.3169	37	0.2327	24	0.3333
AA	29		0.0405	24.72 ± 5.17	2	0.0500	16	0.0360	7	0.0440	4	0.0556
G	1161		0.8108	–	65	0.8125	717	0.8056	267	0.8396	112	0.7778
A	271		0.1892	–	15	0.1875	173	0.1944	51	0.1604	32	0.2222
rs12444979 *GPRC5B*												
CC	524		0.7318	23.99 ± 4.36	29	0.7250	320	0.7191	118	0.7421	57	0.7917
CT	175		0.2444	23.56 ± 4.25	11	0.2750	114	0.2562	37	0.2327	13	0.1805
TT	17		0.0238	23.96 ± 3.79	–	–	11	0.0247	4	0.0252	2	0.0278
C	1223		0.8541	–	69	0.8625	754	0.8472	273	0.8585	127	0.8819
T	209		0.1459	–	11	0.1375	136	0.1528	45	0.1415	17	0.1181

## 2 Experimental design, materials, and methods

### 2.1 Study subjects

From 2009 to 2013, women referred to the Perinatal Centre of the Belgorod Regional Clinical Hospital of St. Joasaph were enrolled. The participants were unrelated Russian women born in Central Russia [11]. Some exclusion criteria were adopted: benign tumors and hyperplastic disorders of the reproductive organs (endometriosis, leiomyoma, and endometrial hyperplasia), malignant tumors of a small pelvis and breast, severe autoimmune diseases, chronic severe diseases of the vital organs (heart, respiratory or renal failure). A total of 716 women met the criteria. This study was approved by the Regional Ethics Committee of Belgorod State University and informed consents were obtained from all participants.

The anthropometric characteristics were obtained by standard methods [12]: a portable stadiometer was used for measurement of height; weight was measured in an upright position, using a calibrated balance beam scale. 5 groups were formed according to BMI: total sample set, sample set with the underweight (BMI<18.50), normal weight (18.50–24.99), overweight (25.00–29.99) and obese (>30.00).

### 2.2 Blood sample collection and DNA handling

Whole blood specimens were collected from each participant using a plastic tubes (Vacutainer®) containing 0.5 M EDTA solution (рН = 8.0) and genomic DNA was isolated from peripheral blood leukocytes using the standard phenol-chloroform method. The isolated DNA was stored at −80°С.

### 2.3 SNP selection

Five SNPs in five genes, rs1514175 *TNNI3K*, rs713586 RBJ, rs887912 *FANCL*, rs2241423 *MAP2K5*, and rs12444979 *GPRC5B* were selected for the analysis according to the following criteria [13]: 1) Previously reported genome-wide and candidate genes associations with body mass index (BMI) and obesity, 2) Regulatory potential (regSNP), 3) Effect on gene expression (eSNP), and 4) Tag value (tagSNP) and 5) MAF > 5%.

The selected loci were associated with BMI in previously published genome-wide and candidate gene association studies (Table 2) and have functional significance: all SNPs appear to have a significant regulatory potential (Table 3) (determined using the online tools HaploReg, v4.1 update November 05, 2015, https://pubs.broadinstitute.org/mammals/haploreg/haploreg.php) and 4 SNPs to influence gene expression level (Table 4) (determined using the GTExportal data, http://www.gtexportal.org/).

**Table 2:** The literature data about associations of the studied polymorphisms with body mass index and obesity.

Chr (1)	SNP (2	Gene/Region	Phenotype	Association (significance) (associated allele/genotype)	Reference
1	rs1514175	*TNNI3K*	**Body mass index**	**0.07 kg/m**^**2**^**(p** = **8 x10**^**−14**^**) (А)**	[1]
			**Body mass index**	**0.06 unit (p** = **3 x10**^**−11**^**) (А)**	[3]
			Body mass index	0.20 kg/m^2^ (p = 7.7 × 10^−4^) (A)	[4]
2	rs713586	*RBJ*	**Body mass index**	**0.14 kg/m**^**2**^**(p** = **6 x10**^**−22**^**) (С)**	[1]
2	rs887912	*FANCL*	**Body mass index**	**0.1 kg/m**^**2**^**(p** = **2 x10**^**−12**^**) (T)**	[1]
			**Obesity (class I)**	**OR** = **1.07 (p** = **1 x10**^**−10**^**) (T)**	[2]
			**Obesity (class II)**	**OR** = **1.1 (p** = **6 x10**^**−9**^**) (T)**	[2]
15	rs2241423	*MAP2K5*	**Body mass index**	**0.13 kg/m**^**2**^**(p** = **1 x10**^**−18**^**) (G)**	
			Obesity	OR = 0.79 (p = 0.029) (A)	[1]
			Body mass index	−0.092 unit (p = 0.028) (A)	[5]
			Children obesity	(p < 0.005)	[5]
			Obesity	OR = 1.34 (0.001) (G)	[6]
			Adulthood body mass index	(p < 0.05)	[7]
			Body mass index	0.11 kg/m^2^ (p = 0.028) (G)	[8]
16	rs12444979	*GPRC5B*	**Body mass index**	**0.17 kg/m**^**2**^**(p** = **3 x10**^**−21**^**) (C)**	[1]
			Body mass index	(p < 0.05)	[9]
			Body mass index (EA)	(p = 0.014)	[10]
			Body fat mass (EA)	(p = 0.002)	[10]

*Note:* The data from the GWAS are shown in bold.

**Table 3:** Regulatory effects of the 5 SNPs of the TNNI3K, RBJ, FANCL, MAP2K5, and GPRC5B genes (HaploReg, v4.1, update November 05, 2015) (https://pubs.broadinstitute.org/mammals/haploreg/haploreg.php).

chr	pos (hg38)	variant	Reference allele	Alternative allele	AFR	AMR	ASN	EUR	SiPhy	Promoter	Enhancer	DNAse	Proteins	Motifs	NHGRI/EBI	GRASP QTL	Selected eQTL	GENCODE	dbSNP
					**freq**	**freq**	**freq**	**freq**	**cons**	**histone marks**	**histone marks**		**bound**	**changed**	**GWAS hits**	**hits**	**hits**	**genes**	**func annot**
1	74525960	rs1514175	A	G	0.31	0.42	0.2	0.56			ESC, ESDR, IPSC			COMP1,ERalpha-a	2 hits	2 hits		FPGT-TNNI3K	intronic
2	24935139	rs713586	T	C	0.9	0.43	0.46	0.46						Pbx3,SP2,TATA	1 hit	3 hits	34 hits	8.5kb 3′ of DNAJC27	
2	59075742	rs887912	T	C	0.94	0.83	1	0.73						Hoxa5,Znf143	3 hits			12kb 3′ of AC007092.1	
15	67794500	rs2241423	G	A	0.4	0.43	0.6	0.23				IPSC			1 hit	1 hit	16 hits	MAP2K5	intronic
16	19922278	rs12444979	C	T	0.09	0.06	0	0.12						LBP-1	1 hit		70 hits	32kb 3′ of AC134300.1

**Table 4:** The *cis*-eQTL values of the 5 SNPs of the TNNI3K, RBJ, FANCL, MAP2K5, and GPRC5B genes (according to Genotype-Tissue Expression (GTEx) (http://www.gtexportal.org/)).

chr	SNP	Gene	Gene expression	Reference allele	Alternative allele	Effect Size (β)	*P*-Value	Tissue
1	rs1514175	*TNNI3K*	*LRRIQ3*	A	G	0.25	0.0000045	Thyroid
2	rs713586	*RBJ*	*ADCY3*	T	C	0.31	0.00000018	Skin - Sun Exposed (Lower leg)
	rs713586	*RBJ*	*CENPO*	T	C	0.71	2.9E-38	Whole Blood
	rs713586	*RBJ*	*ADCY3*	T	C	0.51	5.3E-22	Whole Blood
	rs713586	*RBJ*	*ADCY3*	T	C	−0.23	0.0000000000000063	Nerve - Tibial
	rs713586	*RBJ*	*CENPO*	T	C	0.28	0.00000000000031	Artery - Tibial
	rs713586	*RBJ*	*DNAJC27*	T	C	−0.2	0.00000000012	Nerve - Tibial
	rs713586	*RBJ*	*DNAJC27-AS1*	T	C	0.29	0.0000000003	Whole Blood
	rs713586	*RBJ*	*DNAJC27-AS1*	T	C	−0.27	0.0000000014	Esophagus - Muscularis
	rs713586	*RBJ*	*CENPO*	T	C	−0.39	0.0000000042	Brain - Cerebellum
	rs713586	*RBJ*	*ADCY3*	T	C	0.23	0.0000000091	Artery - Tibial
	rs713586	*RBJ*	*ADCY3*	T	C	0.18	0.000000014	Adipose - Subcutaneous
	rs713586	*RBJ*	*CENPO*	T	C	−0.41	0.00000015	Cells - EBV-transformed lymphocytes
	rs713586	*RBJ*	*ADCY3*	T	C	0.23	0.00000024	Lung
	rs713586	*RBJ*	*ADCY3*	T	C	0.18	0.0000006	Adipose - Visceral (Omentum)
	rs713586	*RBJ*	*CENPO*	T	C	−0.15	0.00000079	Skin - Sun Exposed (Lower leg)
	rs713586	*RBJ*	*CENPO*	T	C	0.18	0.0000011	Adipose - Subcutaneous
	rs713586	*RBJ*	*DNAJC27-AS1*	T	C	0.23	0.0000015	Adipose - Visceral (Omentum)
	rs713586	*RBJ*	*ADCY3*	T	C	−0.33	0.0000026	Pituitary
	rs713586	*RBJ*	*DNAJC27-AS1*	T	C	−0.14	0.000004	Skin - Not Sun Exposed (Suprapubic)
	rs713586	*RBJ*	*ADCY3*	T	C	−0.15	0.000016	Nerve - Tibial
	rs713586	*RBJ*	*EFR3B*	T	C	0.19	0.000031	Lung
	rs713586	*RBJ*	*POMC*	T	C	−0.21	0.000031	Heart - Atrial Appendage
15	rs2241423	*MAP2K5*	*MAP2K5*	G	A	−0.32	4.1E-19	Artery - Tibial
	rs2241423	*MAP2K5*	*MAP2K5*	G	A	−0.4	0.0000000000000029	Heart - Atrial Appendage
	rs2241423	*MAP2K5*	*MAP2K5*	G	A	−0.32	0.000000000000012	Adipose - Subcutaneous
	rs2241423	*MAP2K5*	*MAP2K5*	G	A	−0.27	0.0000000000001	Thyroid
	rs2241423	*MAP2K5*	*SKOR1*	G	A	−0.3	0.0000000000019	Lung
	rs2241423	*MAP2K5*	*SKOR1*	G	A	−0.32	0.0000000000035	Thyroid
	rs2241423	*MAP2K5*	*SKOR1*	G	A	−0.3	0.000000000014	Muscle - Skeletal
	rs2241423	*MAP2K5*	*MAP2K5*	G	A	−0.26	0.000000000015	Whole Blood
	rs2241423	*MAP2K5*	*SKOR1*	G	A	−0.4	0.000000000073	Heart - Left Ventricle
	rs2241423	*MAP2K5*	*SKOR1*	G	A	−0.29	0.00000000021	Adipose - Subcutaneous
	rs2241423	*MAP2K5*	*SKOR1*	G	A	−0.35	0.00000000037	Adipose - Visceral (Omentum)
	rs2241423	*MAP2K5*	*MAP2K5*	G	A	−0.28	0.00000000092	Esophagus - Muscularis
	rs2241423	*MAP2K5*	*SKOR1*	G	A	−0.26	0.0000000014	Artery - Tibial
	rs2241423	*MAP2K5*	*SKOR1*	G	A	−0.23	0.0000000062	Skin - Sun Exposed (Lower leg)
	rs2241423	*MAP2K5*	*MAP2K5*	G	A	−0.22	0.0000000094	Lung
	rs2241423	*MAP2K5*	*MAP2K5*	G	A	−0.29	0.000000011	Artery - Aorta
	rs2241423	*MAP2K5*	*SKOR1*	G	A	−0.25	0.000000019	Skin - Not Sun Exposed (Suprapubic)
	rs2241423	*MAP2K5*	*MAP2K5*	G	A	−0.27	0.000000054	Nerve - Tibial
	rs2241423	*MAP2K5*	*SKOR1*	G	A	−0.2	0.000000095	Nerve - Tibial
	rs2241423	*MAP2K5*	*SKOR1*	G	A	−0.32	0.00000011	Artery - Aorta
	rs2241423	*MAP2K5*	*MAP2K5*	G	A	−0.27	0.00000012	Adipose - Visceral (Omentum)
	rs2241423	*MAP2K5*	*SKOR1*	G	A	−0.48	0.00000013	Pituitary
	rs2241423	*MAP2K5*	*SKOR1*	G	A	−0.3	0.00000013	Breast - Mammary Tissue
	rs2241423	*MAP2K5*	*SKOR1*	G	A	−0.24	0.00000018	Esophagus - Muscularis
	rs2241423	*MAP2K5*	*MAP2K5*	G	A	−0.25	0.00000019	Breast - Mammary Tissue
	rs2241423	*MAP2K5*	*MAP2K5*	G	A	−0.23	0.0000009	Heart - Left Ventricle
	rs2241423	*MAP2K5*	*SKOR1*	G	A	−0.29	0.0000063	Esophagus - Gastroesophageal Junction
	rs2241423	*MAP2K5*	*SKOR1*	G	A	−0.51	0.000011	Ovary
	rs2241423	*MAP2K5*	*SKOR1*	G	A	−0.25	0.000012	Colon - Sigmoid
	rs2241423	*MAP2K5*	*MAP2K5*	G	A	−0.27	0.000013	Esophagus - Gastroesophageal Junction
	rs2241423	*MAP2K5*	*SKOR1*	G	A	−0.2	0.000016	Colon - Transverse
	rs2241423	*MAP2K5*	*MAP2K5*	G	A	−0.38	0.000017	Pituitary
	rs2241423	*MAP2K5*	*RP11-34F13.2*	G	A	−0.29	0.00004	Thyroid
16	rs12444979	*GPRC5B*	*KNOP1*	C	T	0.64	0.00000000000037	Adipose - Subcutaneous
	rs12444979	*GPRC5B*	*KNOP1*	C	T	0.81	0.00000000000000049	Adipose - Visceral (Omentum)
	rs12444979	*GPRC5B*	*KNOP1*	C	T	0.66	0.00000000012	Adrenal Gland
	rs12444979	*GPRC5B*	*KNOP1*	C	T	0.78	0.000000000000000043	Artery - Aorta
	rs12444979	*GPRC5B*	*KNOP1*	C	T	0.67	0.0000000033	Artery - Coronary
	rs12444979	*GPRC5B*	*KNOP1*	C	T	0.43	0.0000026	Artery - Tibial
	rs12444979	*GPRC5B*	*KNOP1*	C	T	0.77	0.0000067	Brain - Cerebellum
	rs12444979	*GPRC5B*	*KNOP1*	C	T	0.89	0.0000000056	Brain - Hypothalamus
	rs12444979	*GPRC5B*	*KNOP1*	C	T	0.82	0.0000009	Brain - Nucleus accumbens (basal ganglia)
	rs12444979	*GPRC5B*	*KNOP1*	C	T	0.81	0.00000000000071	Breast - Mammary Tissue
	rs12444979	*GPRC5B*	*KNOP1*	C	T	0.73	0.0000015	Cells - EBV-transformed lymphocytes
	rs12444979	*GPRC5B*	*KNOP1*	C	T	0.69	0.000000062	Colon - Sigmoid
	rs12444979	*GPRC5B*	*KNOP1*	C	T	0.51	0.0000000005	Colon - Transverse
	rs12444979	*GPRC5B*	*KNOP1*	C	T	0.89	0.00000000000000071	Esophagus - Gastroesophageal Junction
	rs12444979	*GPRC5B*	*KNOP1*	C	T	0.58	0.00000000000000021	Esophagus - Mucosa
	rs12444979	*GPRC5B*	*KNOP1*	C	T	0.75	1.6E-19	Esophagus - Muscularis
	rs12444979	*GPRC5B*	*KNOP1*	C	T	0.73	0.000000005	Heart - Atrial Appendage
	rs12444979	*GPRC5B*	*KNOP1*	C	T	0.56	0.00000035	Heart - Left Ventricle
	rs12444979	*GPRC5B*	*KNOP1*	C	T	0.65	0.00000000000000011	Lung
	rs12444979	*GPRC5B*	*KNOP1*	C	T	0.73	2.00E-20	Muscle - Skeletal
	rs12444979	*GPRC5B*	*KNOP1*	C	T	0.91	0.00000000000000013	Nerve - Tibial
	rs12444979	*GPRC5B*	*KNOP1*	C	T	1	0.000000042	Ovary
	rs12444979	*GPRC5B*	*KNOP1*	C	T	1	0.000000000000029	Pancreas
	rs12444979	*GPRC5B*	*KNOP1*	C	T	0.92	0.000000000000016	Skin - Not Sun Exposed (Suprapubic)
	rs12444979	*GPRC5B*	*KNOP1*	C	T	0.79	4.1E-21	Skin - Sun Exposed (Lower leg)
	rs12444979	*GPRC5B*	*KNOP1*	C	T	0.95	0.000000059	Spleen
	rs12444979	*GPRC5B*	*KNOP1*	C	T	0.67	0.000000000000019	Stomach
	rs12444979	*GPRC5B*	*KNOP1*	C	T	0.55	0.000000000058	Testis
	rs12444979	*GPRC5B*	*KNOP1*	C	T	0.58	0.000000000006	Thyroid
	rs12444979	*GPRC5B*	*KNOP1*	C	T	0.77	0.0000015	Vagina
	rs12444979	*GPRC5B*	*KNOP1*	C	T	0.35	0.000011	Whole Blood
	rs12444979	*GPRC5B*	*GPRC5B*	C	T	−0.25	0.000046	Adipose - Subcutaneous

### 2.4 SNP genotyping

DNA samples were genotyped using the Sequenom MassARRAY® iPLEX platform. The procedure for DNA sample preparation and data quality control are described elsewhere [12].

### 2.5 Statistical analysis

All polymorphisms were checked for their correspondence to the Hardy-Weinberg equilibrium (HWE) using the chi-square test. Differences in allele and genotype frequencies between the groups of underweight (BMI<18.50), normal weight (18.50–24.99), overweight (25.00–29.99), and obese (>30.00) women were determined using the Kruskall-Wallis test.
